# Do Prosthetic Joint Infections Worsen the Functional Ambulatory Outcome of Patients with Joint Replacements? A Retrospective Matched Cohort Study

**DOI:** 10.3390/antibiotics9120872

**Published:** 2020-12-05

**Authors:** Isabel Mur, Marcos Jordán, Alba Rivera, Virginia Pomar, José Carlos González, Joaquín López-Contreras, Xavier Crusi, Ferran Navarro, Mercè Gurguí, Natividad Benito

**Affiliations:** 1 Infectious Disease Unit, Hospital de la Santa Creu i Sant Pau–Institut d’Investigació Biomèdica Sant Pau, 08025 Barcelona, Spain; imur@santpau.cat (I.M.); VPomar@santpau.cat (V.P.); jlcontreras@santpau.cat (J.L.-C.); MGurgui@santpau.cat (M.G.); 2 Department of Medicine, Universitat Autònoma de Barcelona, 08193 Barcelona, Spain; 3 Bone and Joint Infection Study Group of the Spanish Society of Infectious Diseases and Clinical Microbiology (GEIO-SEIMC), 28003 Madrid, Spain; 4 Department of Orthopedic Surgery and Traumatology, Hospital de la Santa Creu i Sant Pau–Institut d’Investigació Biomèdica Sant Pau, 08025 Barcelona, Spain; MJordan@santpau.cat (M.J.); JGonzalezR@santpau.cat (J.C.G.); XCrusi@santpau.cat (X.C.); 5 Department of Microbiology, Hospital Santa Creu i Sant Pau, Hospital de la Santa Creu i Sant Pau–Institut d’Investigació Biomèdica Sant Pau, Universitat Autònoma de Barcelona, 08025 Barcelona, Spain; mrivera@santpau.cat (A.R.); FNavarror@santpau.cat (F.N.)

**Keywords:** prosthetic joint infection, arthroplasty infection, prosthetic joint infection functional outcome, prosthetic joint infection ambulatory outcome

## Abstract

Objectives: To assess the effect on the functional ambulatory outcome of postoperative joint infection (PJI) cured at the first treatment attempt versus not developing PJI in patients with hip and knee prostheses. Methods: In a single-hospital retrospectively matched cohort study, each patient with PJI between 2007 and 2016 was matched on age, sex, type of prosthesis and year of implantation with two other patients with uninfected arthroplasties. The definition of a PJI cure included infection eradication, no further surgical procedures, no PJI-related mortality and no suppressive antibiotics. Functional ambulatory status evaluated one year after the last surgery was classified into four simple categories: able to walk without assistance, able to walk with one crutch, able to walk with two crutches, and unable to walk. Patients with total hip arthroplasties (THAs), total knee arthroplasties (TKAs) and partial hip arthroplasties (PHAs) were analysed separately. Results: A total of 109 PJI patients (38 TKA, 41 THA, 30 PHA) and 218 non-PJI patients were included. In a model adjusted for clinically relevant variables, PJI was associated with a higher risk of needing an assistive device for ambulation (vs. walking without aid) among THA (adjusted odds ratio (OR) 3.10, 95% confidence interval (95% CI) 1.26–7.57; *p* = 0.014) and TKA patients (OR 5.40, 95% CI 2.12–13.67; *p* < 0.001), and with requiring two crutches to walk or being unable to walk (vs. walking unaided or with one crutch) among PHA patients (OR 3.05, 95% CI 1.01–9.20; *p* = 0.047). Conclusions: Ambulatory outcome in patients with hip and knee prostheses with postoperative PJI is worse than in patients who do not have PJI.

## 1. Introduction

Hip and knee replacements are common and increasingly performed surgical procedures. The main indications for total hip arthroplasty (THA) and total knee arthroplasty (TKA) are to relieve pain and improve joint function in patients with advanced joint disease, while partial hip arthroplasties (PHAs) are mostly indicated for restoring function in elderly patients with displaced femoral neck fractures [1,2].

Prosthetic joint infection (PJI) is one of the most dreaded complications of these procedures. Eradication of infection requires surgery and antimicrobial therapy [3,4,5]. Surgical strategies include debridement with implant retention (DAIR) and prosthesis exchange. Cure at the first treatment attempt is critical because each treatment failure worsens tissue damage and functional integrity [6]. On rare occasions, resection of the prosthesis, arthrodesis or amputation is performed to eradicate infection but without restoring full function. Suppressive antibiotic therapy is an option that is not intended to eradicate infection but can minimise symptoms and sometimes preserve function when it is not possible to remove the prosthesis [7].

Unlike other infections, the goal of PJI treatment is not only to eradicate infection but also to relieve pain and maintain joint function; it is not always possible to achieve all these goals [3,4,8]. While PJI treatment success has been primarily defined as eradication of infection [9], few studies have analysed functional outcome, despite this being the main aim of prosthesis implantation. In terms of functional outcome, diverse results have been observed using different surgical procedures for PJI management as compared with uninfected primary arthroplasties [10,11,12,13,14]. The question of whether a PJI cured at the first therapeutic attempt, that is, in the best possible scenario, has a worse ambulatory outcome than an uninfected prosthetic joint, has not been specifically addressed and remains unresolved. Our objective is to assess the effect of postoperative PJI, as compared with not developing PJI, on functional ambulatory outcome in patients with hip and knee prostheses.

## 2. Methods

### 2.1. Setting and Study Design

This study was conducted at the Hospital de la Santa Creu i Sant Pau, a tertiary university hospital in Barcelona, Spain. Patients with PJI are treated by a multidisciplinary team, including medical and surgical specialists.

We used a retrospectively matched cohort study to compare the functional ambulatory outcome of hip and knee arthroplasty patients with (PJI cohort) or without (non-PJI cohort) postoperative PJI.

The Research Ethics Committee of our hospital approved the study.

### 2.2. Study Patients and Controls

Patients with the following criteria were included: (1) a diagnosis of postoperative PJI (excluding haematogenous infections) between January 2007 and December 2016, (2) PJI treatment was intended to eradicate the infection, and (3) the first planned treatment was successful. Since hematogenous PJIs can occur at any time after the index surgery, it would be very difficult to find suitable comparable patients with uninfected arthroplasties to match those with hematogenous PJIs occurring at very different times after prosthesis implantation in order to evaluate ambulatory outcome; for that reason, patients with hematogenous infections were excluded.

Each PJI patient was matched with two control patients with arthroplasties implanted at our institution, who had completed a minimum follow-up of 1 year after surgery without developing PJI. Exact matching was performed on patient sex and age (within a 5-year age range), type of prosthesis (THA, TKA or PHA), primary or revision arthroplasty and year of implantation. Controls for each case were sought by considering all the following patients who underwent the same type of arthroplasty implant; the first two patients who met all the remaining criteria were selected.

### 2.3. Definitions

The diagnosis of PJI was based on International Consensus Meeting for PJI criteria [15]. PJIs presenting within 1 month after surgery were classified as early postoperative infection [3]. When symptoms persisted for more than three weeks beyond one-month postintervention, the infection was defined as chronic [3]. Choice of the optimal surgical strategy for each patient was based on Zimmerli’s algorithm, endorsed by the Infectious Diseases Society of America [3,5]. Despite the one-month postsurgery cut-off used to define chronic versus early PJI infection and the recommendation to remove the prosthesis in cases of chronic PJI, DAIR was allowed up to 3 months after prosthesis implantation, in accordance with Spanish guidelines and recent studies [4,16]. Mobile antibiotic-impregnated cement spacers were used in patients treated with two-stage exchange after removing the prosthesis. Infectious disease specialists selected and controlled antibiotic use. The duration of antimicrobial treatment, based on the Spanish guidelines for the management of PJI, typically ranged from 8 to 12 weeks following DAIR and 4–6 weeks after the first step of a two-stage prosthesis exchange [4]. Infectious disease specialists and orthopaedists followed PJI patients for a minimum of 2 years after ending antimicrobial therapy. Successful PJI treatment (“cure”) was defined following a published consensus definition that included: (1) eradication of infection, characterised by no clinical failure (healed wound without fistula or drainage and painless joint), and no infection recurrence caused by the same organism strain, (2) no further surgical interventions due to infection (other than the one initially planned to treat PJI), and (3) no death caused by a condition directly related to PJI [9]. In addition, suppressive antibiotic therapy was considered a treatment failure. Only patients with PJI cured at the first treatment attempt were included in the current study; the first treatment attempt consisted of the first curative strategy utilised to treat the PJI and comprised a combination of both an appropriate surgical procedure (including DAIR, a one or two-stage prosthesis exchange) and antimicrobial therapy for a definite period of time; patients who required further surgery (such as spacer exchange) or a new course of antimicrobials after the first one ended were excluded. Treatment success was evaluated a minimum of 1 year after ending antimicrobial treatment (for PJIs treated with DAIR or a one-stage arthroplasty exchange) or after reimplantation surgery during a two-stage arthroplasty exchange (with negative intraoperative culture samples).

Under the supervision of a physiotherapist, all patients started full bodyweight bearing ambulation and physical therapy as soon as possible after surgery to facilitate recovery of function. Typically, the rehabilitation program begins from the first postoperative day after TKA and THA implantation (including new prostheses implanted in a one-step exchange or in the second stage of a two-step exchange). After DAIR, inpatient rehabilitation is commonly delayed for a few days (postoperative day 3–5), depending on wound evolution. Exercises to restore normal joint motion and strength are initiated in the hospital and continued upon discharge.

The Charlson comorbidity score and the American Society of Anesthesiologists (ASA) physical status classification system were used to evaluate baseline comorbidities and the patient’s general health status, respectively [17,18].

### 2.4. Ambulatory Outcome

Functional ambulatory status was assessed 1 year after the last surgery. Due to the retrospective nature of the study and the fact that the evaluation of patients with arthroplasties was performed by different surgeons without a uniform scoring system, we classified the patient’s ambulatory outcome in 4 simple categories: (1) able to ambulate without an assistive device, (2) able to walk with one crutch/stick, (3) able to walk with two crutches/sticks and (4) unable to walk. These categories were relative to the patient’s normal outdoor ambulation capacity. Patients with TKA, THA, and PHA were analysed separately.

### 2.5. Statistical Analysis

Continuous variables were summarised as means and standard deviations and categorical variables as percentages relative to the total sample. We used the Wilcoxon and chi-square tests (or Fisher’s exact tests when appropriate) to compare group differences for continuous and categorical variables, respectively. To evaluate whether PJI was an independent factor associated with a worse functional ambulatory outcome, any variable with a *p*-value less than 0.25 in univariate analysis, together with all clinically relevant variables, were included as covariates in an adjusted logistic regression model [19,20]. *P*-values of <0.05 were considered to be significant for all statistical tests. Data were analysed using IBM^®^ SPSS^®^, version 26.0.

## 3. Results

### 3.1. Characteristics of Patients with Prosthetic Joint Infection

A total of 109 patients with postoperative PJI were included: 38 with TKA, 41 with THA, and 30 with PHA. As shown in Table 1, PHA patients were older and more frequently female than TKA and THA patients, who otherwise had similar demographic characteristics. Although most of the patients had early PJI, the percentage was higher in those with PHA. The commonest cause of infection was staphylococci (53.2%) followed by enterobacteria (25.7%). A total of 83 PJIs were treated with DAIR: 81 within the first month after joint replacement surgery (included as “early postoperative infections” in Table 1) and 2 in the second month after index surgery (included as “late chronic infections” in Table 1, in accordance with the above definitions of early and chronic PJIs). Prosthetic exchange was performed on 26 PJI patients (19 two-stage exchanges), 23 of them with chronic infections.

### 3.2. Patients with Infected versus Uninfected Hip and Knee Arthroplasties

Table 2 compares the characteristics and ambulatory outcomes of 109 patients with PJI (cases) and 218 patients without PJI (controls). Patients with PHA (both cases and controls) had more baseline comorbidities and a worse general medical status than those with TKA and THA. In addition, PHA was typically performed to treat hip fractures, whereas total knee and hip replacements were mostly performed for osteoarthritis. Because of these and other well-known differences between patients with PHA and those with THA [21,22,23,24], we analysed them in two separate groups. A detailed comparison of patients with infected versus uninfected TKA, THA and PHA is provided in Table 2. Within each group, patients with and without PJI showed no differences with respect to comorbidity burden and baseline health status, as measured by the Charlson and ASA scores. The indications for joint replacement in PJI and non-PJI patients were similar in the three groups, except for fractures and dislocations, which were more frequent in PJI patients in the THA group (all of these occurred in 7 patients with infected THAs).

### 3.3. Functional Ambulatory Outcomes

**Figure 1:**
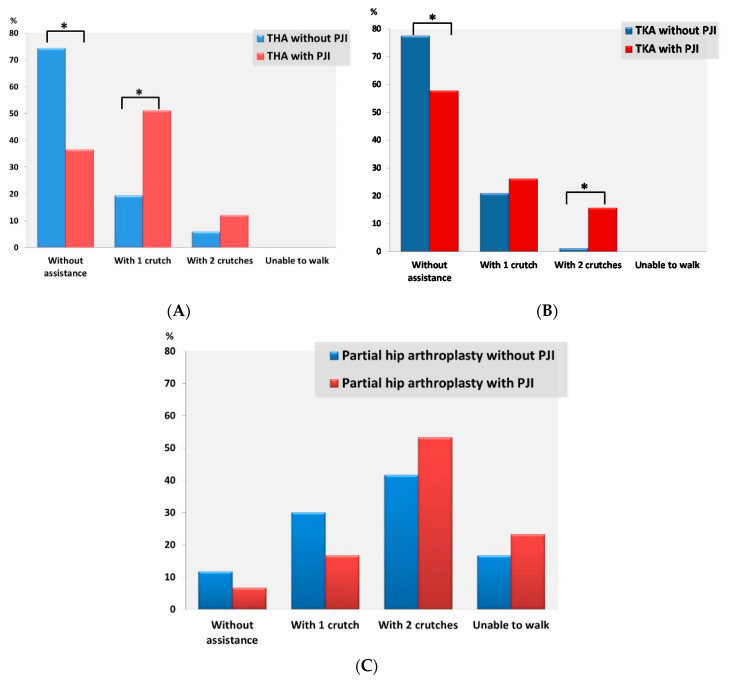
Functional ambulatory outcome in patients with total hip arthroplasty (**A**), patients with total knee arthroplasty (**B**), and patients with partial hip arthroplasty (**C**) with or without postoperative prosthetic joint infection. Total hip arthroplasty (THA), total knee arthroplasty (TKA) and PJI denote total hip arthroplasty, total knee arthroplasty and prosthetic joint infection, respectively. Statistically significant differences are marked with an asterisk (*).

Table 2 and Figure 1 show the functional ambulatory status of patients with and without PJI in univariate analysis.

Most TKA and THA patients (both with and without PJI) were able to walk unaided, but this was significantly more common in non-PJI patients in both the TKA and the THA group; otherwise, patients with TKA and PJI were almost twice as likely to require one or two crutches to walk (42.1% vs. 22.4%, *p* = 0.028), while patients with infected THAs were more than twice as likely to require an assistive device in order to walk than those with uninfected THAs (63.4% vs. 25.6%, *p* < 0.001). With respect to the matched pairs of TKA patients, in which those with PJI were treated with DAIR, differences in walking capacity between PJI and non-PJI patients remained, with non-PJI patients being more often able to walk unaided (79.6%% vs. 59.3, *p* = 0.052) or to walk without assistance or with one crutch (98.1% vs. 85.2%, *p* = 0.040). Regarding matched pairs of THA patients in which PJI patients were treated with DAIR, those with uninfected THAs were significantly more likely to ambulate unaided (77.6% vs. 44.8%, *p* = 0.002). Patients with infected TKAs and THAs treated with prosthesis exchange were more likely to require crutches to walk than their matched pairs of patients with uninfected TKA and THA, although these differences were statistically significant only in the THA group.

Most PHA patients (both with and without PJI) needed two crutches to ambulate. No statistically significant differences were found between PJI and non-PJI patients in the four categories of ambulation capacity, although PHA patients without PJI were more commonly able to walk without assistance or with one crutch, while PJI patients more often required two crutches or were unable to walk (*p* = 0.051). In PHA patients, we further compared their ambulatory ability with that prior to PHA implantation, depending on whether or not they had postoperative PJI, and found no significant differences (*p* = 0.965): (1) 11 (39.3%) PJI vs. 22 (38.6%) non-PJI patients were observed to have the same walking ability as before; (2) walking ability decreased by one stage (e.g., from walking unaided to requiring the help of one crutch) in 9 (32.2%) PJI vs. 11 (36.8%) non-PJI patients; (3) walking ability decreased by two stages in 6 (21.4%) PJI vs. 10 (17.5%) non-PJI patients; (4) a three-stage deterioration was observed in 2 (7.1%) PJI vs. 4 (7%) non-PJI patients.

In patients with TKAs, the adjusted model for clinically relevant variables identified the following factors as being independently associated with a higher risk of needing an assistive device for ambulation (vs. walking without aid): Charlson score ≥2 and PJI (Table 3). Similarly, in analyses of THA patients, older age and PJI were independently associated with a worse ambulatory outcome, defined as requiring an assistive device to walk (Table 4). Considering patients with total hip and knee arthroplasties together, the adjusted model found that older age (odds ratio (OR) 1.07, confidence interval (95% CI) 1.03–1.12), Charlson score ≥2 (OR 3.52, 95% CI 1.45–8.55) and PJI (OR 3.91, 95% CI 2.10–7.37) were risk factors for needing crutches to walk (vs. walking unaided). In the PHA patient group, a worse functional status was defined as requiring two crutches to walk or being unable to walk; in this group of patients, PJI was also identified as an independent factor associated with a worse ambulatory outcome (Table 5). Since there was collinearity between the Charlson score and the ASA classification system used to evaluate the baseline health status of patients, only one of these variables was included in the final adjusted models (Table 3, Table 4 and Table 5).

## 4. Discussion

In patients with hip and knee replacements, we found that having postoperative PJI —even if successfully treated at the first attempt— was associated with a worse functional ambulatory outcome when compared with not having PJI. Patients with total hip and knee prostheses with PJI more often needed an assistive device to walk than patients without PJI. Patients with PHA and PJI were more likely to need two crutches to walk or to be unable to walk than those without PJI.

Although restoring or improving joint function is one of the main goals of joint replacement surgical procedures, few studies have evaluated the effect of PJI on the functional status of patients with hip and knee prosthesis. The studies are heterogeneous, and most of them have important methodological drawbacks that make it difficult to interpret the results. Furthermore, since there are no specific measures to determine functional outcome after PJI [25], different studies have employed a variety of measures, generally extrapolated from those used in total hip and knee arthroplasties. There is also no gold standard outcome measure for arthroplasties [25], which makes it even more difficult to interpret and compare the results of different reports.

Some studies without control groups have evaluated functional outcomes in patients with PJI treated with specific surgical strategies. According to some of them, both DAIR [26] and one-stage arthroplasty exchange [27] showed satisfactory functional results in patients with THA and PJI. A comparison of one- and two-stage arthroplasty exchanges in infected THA patients found better results with the single-stage exchange [28]. Other studies have assessed the functional outcomes of arthroplasty exchanges performed for PJI (septic revision) compared with joint revision surgery performed for noninfectious reasons (aseptic revision) in TKA and THA, with conflicting results. Thus, the results of septic revision were reported to be mostly inferior [29,30,31,32] but also similar [33,34,35,36] and even superior [37] to those of aseptic revision. The variety of indications for aseptic revision in different studies could explain, at least in part, these contradictory results [32].

In recent years, a few studies have evaluated functional outcomes after using different surgical procedures to treat PJI compared with uninfected primary THA and TKA. While some studies showed similar results after PJIs successfully treated with DAIR, as compared with non-PJI patients [10,11,12,14], another one found inferior outcomes in the former group [13]. Overall, the results were worse in PJI patients treated with a two-stage arthroplasty exchange than in uninfected patients [11,12,13,38]. The main limitations of these studies were small sample sizes, functional outcomes evaluated at different follow-up times in PJI and non-PJI patients, and failure to adjust for other variables.

We did not set out to compare the functional results of PJIs according to surgical treatment, since surgical indication is based on algorithms, mainly determined by nonmodifiable circumstances such as time after index arthroplasty [5,16]. Our aim was to assess the influence of PJIs on functional ambulatory outcome in patients with hip and knee arthroplasties, even in the best possible scenario of infections successfully treated at the first therapeutic attempt. This question has never specifically been addressed or resolved. Our study demonstrated a worse ambulatory outcome in PJI than in non-PJI patients one year after the last surgery and after adjusting for other relevant factors influencing the outcome. Due to the well-known differences between patients with PHA and those with THA, we evaluated TKA, THA and PHA groups separately. Although this reduced the statistical power of the total sample size, we found that, in each group, PJI negatively affected the ambulatory outcome of these patients. Furthermore, older age and worse baseline comorbidities were also found to be associated with poorer ambulatory capacity in patients with infected total hip and knee arthroplasties, as previously observed [10]. These factors did not reach the level of statistical significance in the group of patients with PHA, although its smaller sample size limits the value of these results.

Our study has limitations. Firstly, it has the limitations intrinsic to the retrospective design of the study, although it would be difficult and take a long time to find such a large number of patients with PJI and apply the rigorous criteria required in the current investigation using a prospective design. Due to the retrospective study design, we used a very simple scale for functional outcomes focused on walking capacity. More sophisticated outcome measures using quantitative scoring systems have been used in previous studies, some of them specifically for total knee or hip arthroplasties [25]. Although they are a priori more appropriate and precise measures, they also have some disadvantages. First, the heterogeneity of the measures used prevents comparison between studies; furthermore, the clinical interpretation of quantitative measures is not always clear, and statistically significant differences between quantitative measures may not have clinical relevance. Our simple scale of four categories is clinically relevant and easily interpretable. Nevertheless, beyond ambulation capacity, there are other dimensions that are also important when evaluating the results of elective total joint arthroplasties, such as the patient’s quality of life, level of satisfaction and other organ-specific measures [25], but these fall outside the scope of the present study. Furthermore, PJI is an important psychosocial stressor for many patients, which could have influenced their ambulatory outcome, although we could not assess this possibility [39]. Finally, preoperative walking capacity was often not available in the records of patients undergoing total hip and knee arthroplasties, and we cannot, therefore, completely exclude potential baseline differences between infected and noninfected patients. Our study has several strengths. This study evaluating the effect of PJIs on the functional ambulatory result of knee and hip arthroplasties has the largest number of patients. We also evaluated populations that have not been included in previous studies, such as patients with PHA. Finally, our study has overcome some of the methodological limitations of previous studies, making its conclusions more robust.

The results of the present study demonstrate conclusively that having a PJI diminishes the functional ambulatory result that implantation of a hip or knee prosthesis sets out to achieve. It is important to keep this in mind when planning treatment for PJIs and to advise and inform the patient accordingly. These results underscore the need to continue investing effort in the prevention of PJI.

## Figures and Tables

**Table 1:** Characteristics of 109 patients with hip and knee prosthetic joint infection.

Variable	Patients with Prosthetic Total Knee Infection (*n* = 38)	Patients with Prosthetic Total Hip Infection (*n* = 41)	Patients with Prosthetic Partial Hip Infection (*n* = 30)
Age, years—mean (standard deviation)	74 (5.7)	72 (9)	83 (6.1)
Female gender—no. (%)	24 (63.3)	23 (56.1)	26 (86.7)
Primary arthroplasty—no. (%)	33 (86.8)	32 (78)	26 (86.7)
Early postoperative infection—no. (%)	27 (71.1)	29 (70.7)	28 (93.3)
Surgical treatment of early postoperative infections (EPI):			
Debridement and implant retention—no. (% of EPI)	26 (96.3)	28 (96.6)	27 (96.4)
Two-stage exchange no. (% of EPI)	1 (3.7)	1 (2.4)	
One-stage exchange no. (% of EPI)			1 (3.6)
Late chronic infection—no. (%)	11 (28.9)	12 (29.3)	2 (6.7)
Surgical treatment of late chronic infections (LCI):			
Debridement and implant retention—no. (% of LCI)	1 (9.1)	1 (8.3)	-
Two-stage exchange no. (% of LCI)	10 (90.9)	5 (41.7)	2 (100)
One-stage exchange no. (% of LCI)	-	6 (50)	-
Culture-positive prosthetic joint infection—no. (%)	36 (94.7)	38 (92.7)	27 (90)
Microbial aetiology of prosthetic joint infection			
*Staphylococcus aureus*—no. (%)	9 (25)	14 (36.8)	10 (37)
o Methicillin-resistant *S. aureus*—no. (%)	5 (15.2)	2 (5.3)	6 (22.2)
Coagulase negative staphylococci—no. (%)	10 (27.8)	10 (26.3)	5 (18.5)
Enterobacteria—no. (%)	9 (25)	10 (26.3)	9 (33.3)
Other microorganisms—no. (%)	14 (38.9)	10 (26.3)	6 (22.2)

**Table 2:** Characteristics of 327 patients with hip and knee arthroplasties, with and without prosthetic joint infection, matched 1:2 by gender, age ± 5 years, type of prosthesis, primary or revision arthroplasty and year of index surgery.

Variable	Total Knee Arthroplasty (*n* = 114)			Total Hip Arthroplasty (*n* = 123)			Partial Hip Arthroplasty (*n* = 90)		
	PJI (*n* = 38)	Non-PJI (*n* = 76)	*p*-Value *	PJI (*n* = 41)	Non-PJI (*n* = 82)	*p*-Value *	PJI (*n* = 30)	Non-PJI (*n* = 60)	*p*-Value *
Age, years—mean (standard deviation)	74 (5.7)	74 (5.8)	0.936	71 (9)	71 (8.3)	0.817	83 (6.1)	83 (6.0)	0.912
Charlson score ≥ 2—no. (%)	3 (7.9)	11 (15.8)	0.240	4 (9.8)	10 (12.2)	0.772	11 (36.7)	20 (33.3)	0.754
ASA > 2—no. (%)	11 (28.9)	24 (31.6)	0.774	13 (31.7)	28 (34.1)	0.787	17 (56.7)	39 (65)	0.442
Indications for arthroplasty									
Osteoarthritis—no. (%)	32 (84.2)	66 (86.8)	0.703	28 (68.3)	64 (78)	0.240	-	-	-
Aseptic loosening—no. (%)	4 (10.5)	9 (11.8)	1	4 (9.8)	14 (17.1)	0.279	2 (6.7)	3 (5)	1
Periprosthetic fracture—no. (%)	1 (2.6)	1 (1.3)	1	2 (4.9)	2 (4.9)	1	2 (6.7)	4 (6.7)	1
Fracture—no. (%)	0	0	-	4 (9.8)	0 (0)	**0.011**	26 (86.7)	52 (86.7)	1
Dislocation—no. (%)	0	0	-	3 (7.3)	0 (0)	**0.035**	0 (0)	1 (1.7)	1
Rheumatoid arthritis—no. (%)	1 (2.6)	0 (0)	0.333	-	-	-	-	-	-
Functional outcome									
Able to walk without assistance—no. (%)	22 (57.9)	59 (77.6)	**0.028**	15 (36.6)	61 (74.4)	**<0.001**	2 (6.7)	7 (11.7)	0.712
Walking with one crutch—no. (%)	10 (26.3)	16 (21.1)	0.528	21 (51.2)	16 (19.5)	**<0.001**	5 (16.7)	18 (30)	0.172
Walking without assistance or with one crutch—no. (%)	32 (84.2)	75 (98.7)	**0.005**	36 (87.8)	77 (93.9)	0.299	7 (23.3)	25 (41.7)	0.087
Walking with two crutches—no. (%)	6 (15.8)	1 (1.3)	**0.005**	5 (12.2)	5 (6.1)	0.299	16 (53.3)	25 (41.7)	0.295
Unable to walk—no. (%)	0	0	-	0	0	-	7 (23.3)	10 (16.7)	0.446
Functional ambulatory outcome in matched pairs of patients in which those with PJI were treated with DAIR	**PJI treated with DAIR (*n* = 27)**	**Non-PJI (matched with DAIR-treated PJI) (*n* = 54)**		**PJI treated with DAIR (*n* = 29)**	**Non-PJI (matched with DAIR-treated PJI)** **(*n* = 58)**		**PJI treated with DAIR (*n* = 27)**	**Non-PJI (matched with DAIR-treated PJI) (*n* = 54)**	
Able to walk without assistance—no. (%)	16 (59.3)	43 (79.6)	0.052	13 (44.8)	45 (77.6)	**0.002**	1 (3.7)	6 (11.1)	0.415
Walking with one crutch—no. (%)	7 (25.9)	10 (18.5)	0.440	13 (44.8)	10 (17.2)	**0.006**	5 (18.5)	18 (33.3)	0.163
Walking without assistance or with one crutch—no. (%)	23 (85.2)	53 (98.1)	**0.040**	26 (89.7)	55 (94.8)	0.396	6 (22.2)	24 (44.4)	0.051
Walking with two crutches—no. (%)	4 (14.8)	1 (1.9)	**0.040**	3 (10.3)	3 (5.2)	0.396	14 (51.9)	20 (37.0)	0.203
Unable to walk—no. (%)	0	0	-	0	0	-	7 (25.9)	10 (18.5)	0.440
Functional ambulatory outcome in matched pairs of patients in which those with PJI were treated with prosthesis exchange	**PJI treated with prosthesis exchange (*n* = 11)**	**Non-PJI matched with exchange-treated PJI (*n* = 22)**		**PJI treated with prosthesis exchange (*n* = 12)**	**Non-PJI matched with exchange-treated PJI** **(*n* = 24)**		**PJI treated with prosthesis exchange (*n* = 3)**	**Non-PJI matched with exchange-treated PJI (*n* = 6)**	
Able to walk without assistance—no. (%)	6 (54.5)	16 (72.7)	0.437	2 (16.7)	16 (66.7)	**0.005**	1 (33.3)	1 (16.7)	1
Walking with one crutch—no. (%)	3 (27.3)	6 (27.3)	1	8 (66.7)	8 (25)	**0.029**	0	0	-
Walking without assistance or with one crutch—no. (%)	9 (81.8)	22 (100)	0.104	10 (83.3)	22 (91.7)	0.588	1 (33.3)	1 (16.7)	1
Walking with two crutches—no. (%)	2 (18.2)	0	0.104	2 (16.7)	2 (8.3)	0.558	2 (66.7)	5 (83.3)	1
Unable to walk—no. (%)	0	0	-	0	0	-	0	0	-

ASA = American Society of Anesthesiologists; DAIR = debridement, antibiotics and implant retention; PJI = prosthetic joint infection. * Results in bold refer to those that are statistically significant.

**Table 3:** Factors associated with requiring an assistive device for ambulation versus walking without assistance in 114 patients undergoing total knee replacements one year after the last surgical procedure.

Variable	Univariate Analysis			Multivariate Analysis	
	Walking without Assistance (*n* = 81)	Walking with Crutches (*n* = 33)	*p*-Value	OR (95% CI) *	*p*-Value
Age, years—mean (standard deviation)	74 (5.8)	75 (5.5)	0.228	1.07 (0.98–1.16)	0.120
Female gender—no. (%)	48 (59.3)	24 (72.7)	0.176	1.90 (0.75–4.81)	0.175
Charlson score ≥ 2—no. (%)	8 (9.9)	7 (21.2)	0.129	**3.94 (1.15–13.53)**	0.029
ASA > 2—no. (%)	23 (28.4)	12 (36.4)	0.403		
Revision arthroplasty (versus primary arthroplasty)—no. (%)	11 (13.6)	4 (12.1)	1	0.906 (0.25–3.28)	0.881
Urgent surgery (versus elective surgery)—no. (%)	2 (2.5)	0 (0)	1	-	-
Postoperative prosthetic joint infection—no. (%)	22 (27.2)	16 (48.5)	0.028	**3.10 (1.26–7.57)**	0.014

ASA = American Society of Anesthesiologists; CI = confidence interval; OR = odds ratio. * Results in bold refer to those that are statistically significant.

**Table 4:** Factors associated with requiring an assistive device for ambulation versus walking without assistance in 123 patients undergoing total hip replacements one year after the last surgical procedure.

Variable	Univariate Analysis			Multivariate Analysis	
	Walking without Assistance (*n* = 76)	Walking with Crutches (*n* = 47)	*p*-Value	OR (95% CI) *	*p*-Value
Age, years—mean (standard deviation)	69 (8.4)	74 (7.8)	0.001	**1.10 (1.03–1.16)**	0.003
Female gender—no. (%)	41 (53.9)	28 (59.6)	0.541	0.97 (0.39–2.45)	0.949
Charlson score ≥ 2—no. (%)	6 (7.9)	8 (17)	0.121	3.02 (0.76–12.01)	0.116
ASA > 2—no. (%)	22 (28.9)	19 (40.4)	0.189		
Revision arthroplasty (versus primary arthroplasty)—no. (%)	13 (17.1)	14 (29.8)	0.099	1.04 (0.34–3.21)	0.942
Urgent surgery (versus elective surgery)—no. (%)	3 (3.9)	10 (21.3)	0.005	3.41 (0.66–17.70)	0.145
Postoperative prosthetic joint infection—no. (%)	15 (19.7)	26 (55.3)	<0.001	**5.40 (2.12–13.67)**	<0.001

ASA = American Society of Anesthesiologists; CI = confidence interval; OR = odds ratio. * Results in bold refer to those that are statistically significant.

**Table 5:** Factors associated with requiring two crutches to walk or not being able to walk versus walking without assistance or with one crutch in patients undergoing partial hip joint replacements one year after the last surgical procedure.

Variable	Univariate Analysis			Multivariate Analysis	
	Walking without Aid or with 1 Crutch (*n* = 32)	Walking with 2 Crutches or Not Able to Walk (*n* = 58)	*p*-Value	OR (95% CI) *	*p*-Value
Age, years—mean (standard deviation)	81 (6.0)	84 (5.7)	0.010	1.08–0.99 (1.18)	0.068
Female gender—no. (%)	27 (84.4)	51 (89.9)	0.748	1.02 (0.26–4.06)	0.973
Charlson score ≥ 2—no. (%)	7 (21.9)	24 (44.4)	0.062		
ASA > 2—no. (%)	31 (59.6)	41 (70.7)	0.026	2.59 (0.96–7.01)	0.062
Revision arthroplasty (versus primary arthroplasty)—no. (%)	5 (15.6)	7 (12.1)	0.748	2.75 (0.27–28.13)	0.393
Urgent surgery (versus elective surgery)—no. (%)	28 (87.5)	57 (98.3)	0.052	22.29 (0.77–641.97)	0.070
Postoperative prosthetic joint infection (versus uninfected arthroplasty)—no. (%)	7 (21.9)	23 (39.7)	0.087	**3.054 (1.01–9.20)**	0.047

ASA = American Society of Anesthesiologists; CI = confidence interval; OR = odds ratio.* Results in bold refer to those that are statistically significant.
